# New York State Veterinary Medical Society

**Published:** 1893-11

**Authors:** 


### SOCIETY PROCEEDINGS.

### PROCEEDINGS OF THE FOURTH SEMI-ANNUAL MEETING OF THE NEW YORK STATE VETERINARY MEDICAL SOCIETY.

The fourth semi-annual meeting of the New York State Veterinary Medical Society was held at the Genesee Hotel, Buffalo, N. Y., on Wednesday and Thursday, Oct. 11th and 12th, 1893.

*First Day - Morning Session.* In the absence of Dr. Huidekoper, the President, from illness, the meeting was called to order by the Vice-President, Dr. Wende, at 10.15 A. M. Upon roll call the following members were present:

Drs. John A. Bell, J. M. Chase, Baker, A. Drinkwater, W. Huff, N. P. Hinkley, E. D. Hayden, C. D. Morris, L. A. Robinson, H. Sutterby, F. Sutterby, J. K. Sutterby, E. A. Wieland, John Wende, J. A. Genung, George E. Gangloff, J. M. Pendergast, E. J. McLeod, S. Somerville, Jr., G. G. Dean, E. Pohl, Bell, Erie, Pa., W. Horace Hoskins, Philadelphia, and Prof. Richardson, Toronto, Ont.

New members present:—R. H. McMullen, Jas. W. Darby, B. P. Wende, J. P. Thompson, Anderson Cromforth, W. H. Kelley, H. D. Martin, D. E. Block, and C. J. Willganz.

The Vice-President then gave the following address:

Gentlemen:—For the second time I have the honor of assisting in opening the proceedings of this Society, added to which I have the pleasure of welcoming you to our city. I trust that this session will prove as beneficial, instructive and pleasant as those which have preceded it. The increased attendance shows the growing interest and importance of our organization, and it is with no small degree of pride to each one of us to see this evidence of our professional advancement and importance in the community.

It is within the recollection of many of us when it would have been beyond our anticipations to look forward to the present era of veterinary education, practice, ethics and organization, and our rapid development should act as a stimulus to promote its welfare in every way. Our craft was formerly self-educated, if educated at all; our therapeutics much like that of the Chinese of to-day; colic was treated with chickens' guts, or the abdomen was kneaded with a No. 10 boot, or rubbed with a fence rail. Ethics were unknown—probably few could have spelt the word—while organization was never considered.

The importance of our profession to the community is now established in many directions, notably the inspection of cattle, meat, food, and the recognition and stamping out of contagious diseases, are but few of the important duties which now devolve upon us. There is probably no professional field which presents more opportunities for original work than ours does to-day, and particularly in pathology and surgery. The application of the principles of antiseptic surgery as applied to human beings may be modified to suit our own practice.

The time also seems to be present when some effort should be made toward the education and licensing of horseshoers. The man who can improve the present system of shoeing horses so that it will be more durable and less destructive to the feet, particularly in large cities with the new asphalt pavements, will probably have not only fame but fortune as his reward, and instances like this might be multiplied. We are glad to see you in Buffalo, which is a fitting place for veterinarians to meet, for we are in the centre of some of the most celebrated stock farms on earth. In closing I wish you all a joyful and interesting time during this convention.

The Assistant Secretary, Dr. Drake, read an abstract of the minutes of the last annual meeting.

*Secretary Hinkley.*—I hope that you will pardon this report of our last proceedings. We took all precautions to have a very good report, and employed a stenographer, that I supposed was a first class man; he was so recommended. But unfortuately, after he had taken a very little of the proceedings, he became so intoxicated that he did not know what he was doing, and it was left to a long hand and short hand—a complicated affair, and it is a garbled up proceedings, as you see. This time we have a man that will give us a satisfactory report of present meeting.

*The Chair.*—The next in order will be the receiving of applications for membership and I will declare a recess for that purpose. (A recess was then taken for receiving applications for membership.) In the absence of four members of the Board of Censors, I will appoint Drs. Sutterby, Huff, Robinson, and Claud D. Morris to act as Censors *pro tem* at this meeting.

Meeting called to order.

*The Chair.*—Gentlemen, the Board of Censors have acted favorably upon the following applications for membership to our Society:

New members elected:—R. H. McMullen, Buffalo: Jas. W. Darby, Fort Plain; B. P. Wende, Buffalo, Anderson Cromforth, Lockport; J. P. Thompson, Niagara Falls; W. H. Kelley, Albany; H. D. Martin, Buffalo; D. Eugene Block, Buffalo; C. J. Willganz, Buffalo; L. G. Moore, Trenton, N. Y.
The election of these applicants is now ready for your consideration.

*Dr. Bell.*—I move that these applicants be accepted as members of our Society. Carried.

*The Chair.*—The next order of business is the introduction of new members. It will be necessary to do this as quickly as possible, and if the new members will please rise to their feet as their names are called, I think that will be sufficient. The chairman then introduced to the meeting the members elect, with the exception of Drs. Moore, Block and Kelly, who were absent.

*The Chair.*—The next in order is unfinished business.

*Secretary Hinkley.*—At our last meeting there was some discussion on the proposition of changing the regular place of meeting of this Society, which, as you all know, has heretofore been Syracuse, and the adoption of the plan of the United States Veterinary Medical Association, and having yearly or semi-annual meetings at whatever city in the State of New York will give us the most advantages and inducements to go there. That was not finished, and I think would be one of the essential things to discuss while we are here, whether to continue our meetings at Syracuse as heretofore, or to change our place of meeting every year; also change from semi-annual to annual meetings. I think that is something that you are all interested in this morning. It certainly is very important.

*Dr. Bell.*—I for one am in favor of changing the meetings from semi-annual to annual, and also changing the meetings to different places according to the vote of the members present at any meeting. I think that is a question that should be voted upon by the members in attendance at the preceding meeting, the location of the next ensuing meeting.

*Dr. Morris* —There is considerable ground for remarks on our present by-laws in that respect. We located at Syracuse for the reason that it was central, easy of access, and perhaps was a point that would be favorable for exciting a little interest and bringing the members of the profession together whenever the Society was in session. Inasmuch as we have been to Syracuse on two or three occasions, and with the exception of one gentleman in the profession there, perhaps three or four in the city practicing who are legally authorized to practice, we were not recognized by the local profession at all, and they did not show us the courtesy that we had a right to expect from them. I think it is a good plan to go from city to city each year, and induce in that neighborhood and vicinity an acknowledgment from the profession. It would build up the membership, and it would incite a better feeling; we should be profited by it. As to the semi-annual meeting, I think it is wise from our past experience to discontinue it, for the simple reason that it seems to be a pretty hard matter to get gentlemen to leave their business and come to even a one or two days' meeting at the most a two days' meeting; and if we can induce them to come once a year and have a two days' meeting, it would seem to me to be far more profitable than meeting twice a year and have a one day's meeting on each occasion. It seems to me that there is much in favor of changing the place of meeting, and having it determined at each preceding meeting, as Dr. Bell has suggested, where the succeeding meeting shall be held, and especially in favor of discontinuing the semi-annual meeting. I for one am very much in favor of the change.

*The Chair.*—We have here Dr. Hoskins of Philadelphia, Secretary of the United States Veterinary Medical Association, and I should be pleased to have him express his views on this subject. He certainly has had a wide experience.

*Dr. Hoskins.*—I thank you, Mr. President, for the recognition. I do not know, I am sure, whether I am qualified to express an opinion in New York State on this subject. In our own State, Pennsylvania, where I have the honor of being President of the State Association, we still cling to the two meetings a year, but we have there, as you have had in New York State, a fixed place for our annual meetings. It is always in Philadelphia, though, as you are aware, that is at the extreme eastern part of the State. Still, New York State may be so large, and your veterinarians may be so widely scattered—and I was somewhat impressed with that point this morning in riding into Buffalo over the Lehigh Valley, the stations being so wide apart—that it might be better in New York State for you to have but one meeting a year; and of course, in the event of adopting but one meeting in a year, I should certainly advise that it be left open for the meeting to discuss and decide. In Pennsylvania, where our semi-annual meeting is always held outside of the city of Philadelphia, there is always quite a rivalry by the various cities in the State to secure the meeting at their place, and we are always most royally entertained by the veterinarians at those respective towns. We have for years tried to encourage the development of local associations wherever we could in the State. If there were ten or twelve veterinarians in three or four adjoining counties, we tried to prevail upon them to organize a local association, and to work in conjunction with our State Association; and, while it rather ill becomes me to say it, we are very proud of our State Association in Pennsylvania, and we have had a career that has been unbroken, so far as progress is concerned, and I might say that that has largely resulted from great care in the selection of officers. And in talking to the gentleman on my right here, in reference to another matter, the question of your Board of Censors, or Comitia Minora, came up, and I feel that that is a most important part of a veterinary organization to have a strong body of men on your Board of Censors, men that will try to elevate the profession and lead it forward. If I were a member of your State Association, and found that semi-annual meetings were not a success—ours in Pennsylvania are a success ; we never have had one that was not better than the one preceding—if, as I say, I were in New York, and felt that the semi-annual meeting was not a success, the State being so large in territory, I should certainly advise doing away with the semi-annual meeting, and holding one of two days annually. There is also another question to be considered in connection with the number of meetings you hold, if your State is large and the territory extensive. The consumption of time of veterinarians twice a year must always be taken into consideration, and in your case probably one meeting a year would be better than two. You would get a larger attendance, you would get more interest, and if it were fixed at a time of the year, as it could be, when veterinarians would want to take a vacation, they could include this as a part of their trip.

*Secretary Hinkley.*—I would like to ask Dr. Hoskins whether, in the United States Association, it is left discretionary with the Comitia Minora to choose the place of meeting, or whether there is a general vote taken by the members, or who has authority for fixing the place of meeting?

*Dr. Hoskins.*—The Comitia Minora has the power to fix the time and place, although before closing the United States Veterinary meetings the matter is always brought up by some one, and if any petitions are sent in or an expression of opinion given there for the guidance of the Comitia Minora, it is generally followed by them. As you will remember, it is only three years since the United States Association did away with their semi-annual meeting. At that time they rotated between New York and Boston, the annual meeting always being held in New York and the semi-annual in Boston. Well, it was a very unwise part of their programme to have two meetings a year, so that was done away with, and the annual meeting was established, and the change added wonderfully to the success and the growth of the organization. But the Comitia Minora always give notice of the time when they propose to hold a meeting, which is called for by the by-laws once in six months, and give due notice to the members that a meeting will be held for the purpose of deciding the place of the next annual meeting, and that gives an opportunity for the veterinarians of any city or State organization to send in any petition or to send any delegate there to represent them, and hold out such inducements to the organization to go there as they desire ; and I remember it was only at our meeting about a year ago when Buffalo sent in a strong petition to come here. I had the pleasure of voting for it, and I also had the pleasure of being whipped. Still I think Buffalo would be a very suitable place for a meeting of the United States Association, and I might throw out a suggestion here that some action on the part of your organization to-day or to-morrow might lead to the securing of the meeting in '94, as that has not been fixed in any sense of the word, and of course it will come East. The '94 meeting will be held in the East. The organization now proposes to hold the meetings so as to rotate between the East and the West, one year in the East and the next in the West. But, after all, it is fixed by the Comitia Minora.

*Dr. Sutterby.*—Mr. President, you remember what my views were in Syracuse last winter. I was much in favor of having the annual meeting and dispensing with the semi-annual meeting ; and, as has been proposed here to-day, to determine by a vote at the annual meeting the place where the following meeting shall be held. That is a point well taken. I endorse it, and believe that as far as diffusing fellowship and good feeling, it will have that tendency throughout the country—to change the place of meeting. As has been remarked Syracuse did nothing for us in the way of receiving or entertaining the Society, and there was not but the attendance of one, if I remember, of the local veterinarians at that place. I think the minutes of the meeting will show that. I should be in favor of the annual meeting, and leaving the choice of place not to the Comitia Minora or Censors, but to a discussion in open meeting and a vote of the members present.

*Dr. Huff.*—I am in favor of the annual meeting and the change of place as the rest of the members are I think it would be a good plan for the veterinarians of the different cities to decide what they would be willing to do for the Society and report at the meetings, and in that way we will hear from all the cities, and ascertain the desired information about rates, &c.

*Dr. Sutterby.*—I would move that the semi-annual meetings of this Society be discontinued. Seconded. Carried.

*Dr. Morris.*—I move that in future it shall be the duty of the Board of Censors to fix the date and place of the succeeding meeting of this Society, and that this provision shall be engrafted in the by-laws.

Now, in making this motion, I want it understood to be a little broader than the motion really implies, that while the Board of Censors shall entertain petitions or suggestions, or whatever may be said in open session regarding the future meeting, and shall take into consideration all the circumstances, yet I think it is essential that the Board of Censors should control this matter. It seems to me that it ought to be left more to a body of men, or to two or three men, after hearing all the pros and cons on the subject, and that they could decide better and more in the interest of the Society.

*Dr. Sutterby.*—I second Dr. Morris' motion, and, further, would be in favor of an amendment to Dr. Morris' motion, providing that the place of meeting should be decided at the previous meeting, so that every one present could have a vote upon the question He thought that method would avoid any feeling or antagonism, while if there were then any inducements that the members of the different localities desired to hold out to the Association, they would be recognized by the meeting, and the question decided at the meeting.

This amendment was seconded by Dr. Bell of Watertown.

*Secretary Hinkley.*—This is an important question, and one that every member in the house ought to take some interest in. Now is the time to speak. Do not wait until after it is voted to make the change before making known your objections, if you have any. Now is the time and place to speak, and I for one feel as though we cannot take any too much time in the discussion of our by-laws, if we don't do anything else. Vote just as you feel. I know I am going to.

*Dr. Hoskins.*—If you will allow me to make a suggestion, it would be better for the by-laws to fix the date. I would make that a fixed time every year. I would be careful to have it so arranged that it would not be in conflict with any other meetings, that are already fixed by State organizations adjacent to you, like the Pennsylvania, the New Jersey, and likewise the United States Association. Then it is always a simple matter of reference, and is not difficult for any member to consult his by-laws and find out just the date of the meeting exactly. It is a fixed date. If it was the second Tuesday or the first Tuesday in September, or the third Tuesday in August, he could always fix that time. He would not have to conuslt any one, or lose the time necessary in writing to the Secretary or any other officer.

*Dr. Morris.*—The old by-laws already fix the date of the annual meetings.

Secretary Hinkley then suggested that some of the members preferred to visit the stock farms of Mr. Hamlin or Jewett rather than to take the proposed excursion to Niagara.

*First Day—Afternoon Session.*

The meeting was called to order at 4.30 P.M., Vice-President Wende in the chair.

*The Chair.*—The order of business now open for your consideration is new business.

*Dr. Morris.*—Under the head of new business I have a motion that I would like to submit. I move you that inasmuch as some very important information of a questionable nature is at hand regarding two institutions pretending to teach veterinary medicine in this country, and that whereas three gentlemen have asked admission into our Society who are graduates of these institutions, this Society reconsider the admission of these candidates, and that they be not balloted for at this meeting, but shall go over until the next regular meeting of the Society.

The institutions in question are the Ohio Veterinary College at Cincinnati, and the New York Veterinary College in the City of New York. The Ohio College, I think, was established two years ago this present month. The gentleman—if I can call him a gentleman—appropriates to himself the title of "Dean" of this institution. We have all been educated to look upon the term "dean" as being one very specific in character. However, that is immaterial to the question; it only goes to show the gravity of the case and the depth of degradation into which this institution has already been dragged. The term "dean" applies to a man recognized as a sage in an institution of learning, and is a degree conferred by some institution or university of established reputation, composed of men of the highest ability and of unblemished character. This man, A. H. King, who styles himself "Dean" of the Ohio Veterinary College, was kicked out of the Toronto Veterinary College simply because he was a man who was given to all vice known almost in that city, and it was a disgrace to that institution to tolerate him any longer, and he goes to Cincinnati, an alien, not an American citizen, and establishes a school for the sole purpose of putting through students who are not able to pass the regular examinations at Toronto. He does this out of malice toward the Toronto school; and in anywhere from six days to six weeks they have a certificate from that institution alleging to qualify them for practice. Now we have enough to contend with in our profession without having to contend with any such institution as that; and as we started out only two or three years ago to build up this Society on merit, I think we should very carefully guard at the present time and in future against the admission of members from such schools as that. The school in New York City—and I can vouch for all I say—has for its main man a person who sold a diploma for fifty dollars. The party that he sold it to told me about it. I have seen the diploma. He gave his note for it, and it is'nt paid yet. He sold another one to another man man for ten dollars. Now, we do not care for members holding certificates from such schools as that. We don't want them; they are no benefit to us; they are a disgrace to the profession. We tirade a great deal about quacks, but I can bring more veterinary surgeons, with sheep skins, that will disgrace our profession than I can quacks that are a disgrace to our profession. Of course the profession was primarily dragged down by them, but to-day it is being dragged down by a different class of men. Now, in the light of this information I ask for the adoption of this motion. It can be done in perfect harmony with parliamentary rules, with the consent of the Society. I am not speaking in the least derogatory of these applicants ; they are perfect gentlemen, so far as I know, and should be recognized perhaps. I refer to Dr. Pohl, of this city, of the Ohio Veterinary College; Dr. Wm Henry Kelly of Albany, of the New York School of Veterinary Medicine; and Dr. Modinger, who signs himself "Professor" C. F. Modinger, Professor of Surgery of the New York School of Veterinary Surgeons, and is vouched for by a very reputable member, too, two very reputable members of our Society, Roscoe Bell and George H. Burns, both of Brooklyn. I know those gentlemen personally ; they are very fine men and well qualified men, but it only goes to show how little regard is shown for the responsibility which rests upon them in vouching for members coming from such an institution. Now, they despise that institution just as much as I do, knowing the same facts that I do. With this light I think we should consider well whether or not it is to our advantage to admit men from such institutions into our Society. However, I should like to hear something from the other members on this subject. If I am wholly in the wrong, or too cranky on this matter, why then I should be over-ruled.

In compliance with a request from the chair Dr. Morris again read the motion offered by him.

*The Chair.*—Gentlemen, this certainly is a question of some importance. It should not be acted upon hastily. It should be considered duly, and I should like to hear the fullest expression of the views of all members upon the subject. As for my part I quite coincide with what Dr. Morris says so far as I am acquainted with the facts. I am acquainted with the facts in regard to Dr. King of the Ohio Institution. As to the other institution I am acquainted with no one connected with that.

*Dr. Morris.*—I would like to say another thing in relation to the New York School, for the information of the Society, and it is this. Dr. Gill who is the only veterinarian connected with that school, is the Dean, he styles himself Dean also, and all the other professors are regular medical men, which, while of course it is not wholly irregular is nevertheless so to a certain extent. A veterinary school should be taught and instructed by veterinarians, not by medical men. The characteristics of disease existing in the higher and the lower animals are quite distinct in many instances, and require distinct and specific treatment. A medical man does not go about finding out all the pathology of the characteristics of diseases of the lower animals ; he has something else to attract his attention and engage his mind. So far as the teaching of anatomy is concerned, he can only do it in a comparative way, and so far as physiology and chemistry are concerned, why that he can do in a perfunctory way ; it does not require any very great amount of veterinary skill for an M.D. to teach that. Then again, this school last winter got into a personal fight among themselves. They have voted Dr. Gill* entirely out of the institution, and it is now run wholly by M. D.'s, for but one purpose—to make money. They do not care that for the veterinary profession; they laugh at the very idea of trying to build up a veterinary profession. They say themselves they are in for a money scheme. Now, we don't want members in our Society who come here purely for the sake of advertising themselves as members, or who come purely out of the feeling that they are going to make money out of it. That isn't what we are here for ; it is for building up the profession in this State, and we can only hold to build it up by bringing into our Society men of character, and who come from institutions of recognized character. While I admit that one or two of the gentlemen were taken in last January, they have not received their certificates of membership yet ; and I am glad of that, because we can easily refund them their dues and state the reasons

* There is some error in Dr. Morris' statement ; Dr. Gill has been continuously and is now connected with the New York College of Veterinary Surgeons, and several other veterinarians are associated with him in the corps of instructors. [Ed.]

why we cannot accept them if it is necessary. But I tell you it behoves us to go slow and sure. Let us do our part in our day and generation, that whoever is fortunate enough to come after us in this Society will be glad that we stood by a few principles of right, which I think it is our duty to do as veterinarians.

*Dr. Somerville.*—I would like to ask Dr. Morris if he has ever heard of any diplomas being sold by the Cincinnati Veterinary College?

*Dr. Morris.*—No.

*Dr. Somerville.*—Or any graduate that has become a disgrace to the profession?

*Dr. Morris.*—From the Ohio Medical College?

*Dr. Somerville.*—Yes.

*Dr. Morris.*—I base my remarks wholly upon the man who runs the institution. It is a snide institution from beginning to end; nothing else.

*Dr. Somerville.* I understand that he is out of that.

*Dr. Morris.*—I can tell you how perhaps he may be out of it. He got into a drunken brawl in the streets of Cincinnati last summer, and he was pretty nearly beaten to death, and it is possible it has laid him up so that he is not in it at present. However, he is the man that established the institution, and he has about him a class of men of nearly equal calibre. I have made investigation regarding that school. It is a snide school, and I hold it up for any man to investigate, and when he has done so he will throw up his hands the fumes will be so obnoxious.

*Dr. Somerville.*—I simply asked for information.

*Dr. Morris.*—Certainly; I am willing to give all the information I can. But as regards the school in New York, I have seen, as I said, one of the gentlemen who shows me the certificate he bought for $50.00, and he braggs about it; and he told me of another man, pointed him out to me, and said "There goes a gentleman who bought one for ten dollars;" and I suppose he braggs about that.

*Dr. Bell*, of Watertown.—Mr. President, I quite agree with Dr. Morris. I think there ought to be a line drawn, although it will probably require a little changing of the by-laws on that one subject. I am quite in favor of drawing the line there. It is the only way we can put a damper on such institutions. I am proud to second that motion.

*Secretary Hinkley.*—I would like to ask for a little more time, not that I am either pro or con on this subject, but I think it is a grave subject, and one that every member here should take some part in the discussion of. Now we have taken in these members: we have gone through our usual form of investigation, and we have voted to accept them, this morning, as members. Of course that can be all rescinded the money con be returned to them; but we have heard only the voice of three or four in this room. There are others that certainly have an opinion. If they are lukewarm on this subject, and there are only two or three of us that want these colleges barred, we certainly cannot call it the sense of the meeting. I say this out of no personal regard for either college, but I would like to see the question a little more thoroughly discussed. We have men here that are able to do it, and who must feel either for or against these colleges. Let us have a free and full discussion; do not let us hurry this thing through.

*The Chair.*—Dr. Sutterby, will you favor us with your views on this subject?

*Dr. Sutterby.*—If Dr. Morris can substantiate what he says I am in favor of rescinding the action taken this morning relative to the men elected from those colleges.

*Secretary Hinkley.*—I would like to enquire if either of the gentlemen referred to is in the room?

*Dr. Kelly*, of Albany.—I am one.

*Secretary Hinkley.*—Why not hear Mr. Kelly's side?

*Dr. Kelly*,—This is news to me. The Doctor makes the statement that Dr. Gill was the only veterinarian connected with the college. That I cannot say for since I was graduated, but at the time I attended, there were Dr. L. McLean of Brooklyn, who is an old country graduate, as Professor in Cattle pathology, Dr. R. McLean, Prof. James Hamill.

Dr. Heard of New York City, an M. R. C. V. S.; there was Dr. Critchison, an M.D and V.S., who had charge of the Fourth Avenue car stables at the time, and Dr. McCaffrey, Dr. Gill, and I think one or two others, all veterinarians; while at the same time we had as M. D. professors Dr. Herman M. Biggs, Dr. Grower, Dr. Reuling, Dr. Knight; and Dr. Michener was also connected with the college when I was there He is a V. S., and Dr. Drake, who is an M. D. and V. S. As to what has been since that time, I know nothing about it. It is four or five years since I was graduated. At the same time I am in favor of holding up the professional standard, and if there is going to be any question at all, I would prefer to withdraw my application if it can be done,—not but that I send it in all good faith; but that is not that,—it is the interests of the profession that are paramount. It is the interests of the society hereafter that are entitled to consideration. I know since I left Prof. Hamill has left and the two McLeans, Dr. Michener and Dr Heard have left. I know a number of veterinarians left the year after I was graduated, from some trouble in the faculty. But it is news to me that any one ever bought a diploma.

*Dr. Morris*,—I am very glad that Dr. Kelly is here and has had the courage to stand up in behalf of his interests. He has that right, and I am myself convinced that the institution when it first started out, had good intentions, but as the Doctor himself says, there was some trouble in the faculty some three or four years ago. Perhaps some of their best men were thrown overboard.

*Dr. Kelly*,—The best men have gone; what were considered the best men in the college have all gone; that I know.

Dr. Morris made further charges, that diplomas were sold for $50 and for $10 and suggested that it be investigated by the Board of Censors. He wished for the reconsideration of the election of Drs. Kelly, Pohl and Moadinger.

*Dr. Hoskins.*—I do not know that this is a matter that I ought to take any part in. It is confined rather strictly to your Society, but I certainly feel that you are treading on extremely dangerous ground; I certainly feel that you are doing a very questionable act; and it is a question in my own mind as to whether it is a legal act for you to take up these names now, that you have balotted for, and rescind their election in the way that you propose to do it. You propose to re-consider their election, which in reality was final. I can see the unfortunate position you are in, and can appreciate it fully; but it seems to me that you are going at it in a way that will establish a bad precedent. My own idea of handling such cases as that would be, rather, to have referred the matter to the Board of Censors in its entirety, with instructions to investigate; and certainly you would not have fallen into one error which you have already discovered that you have fallen into, of placing one of these men under the ban of this severe criticism, unjustly. There is no question that one of these applicants graduated from this school at a time when it was in good standing, and when it had a corps of efficient professors, capable of properly educating him for the veterinary profession, and yet you place him under the ban of this criticism with the others. It seems to me that a method that I should have advised if it had been in our own Association, or in the United States Association, would have been to refer this matter to the Board of Trustees or Board of Censors, and then to have taken these names up specifically and reconsidered them as having been elected under a misapprehension, under a lack of knowledge of the condition of affairs that was existing in the schools that had sent them forward. I think that neither school in its present condition should be recognized.

At the time the school started out and offered to graduate minors, that was one of the first inducements that they offered. All that has been said by Dr. Morris in regard to the gentleman is only too true, I am sad to say. It has pained me more than a little to notice in the papers throughout the Associated Press wherever it has reached, this information relative to the Dean of that school. It is only too true that that school was started solely for commercial purposes, and had no other object; it is only too true that they have never done anything as a school to encourage or to advance the veterinary profession to a higher standard, nor do they show any evidence of it; nor has the New York Veterinary College since the dissension there ended in the dismissal from that institution of the professors that have been named by Dr. Kelly of Albany.

I notice one other point in connection with your applications for membership that we should not allow in our Association in Pennsylvania, nor is it now allowed in the United States Association. As far as possible all applicants' names are printed on the programme, together with the name of the college from which they are graduated and their vouchers. That is sent to each member, insofar as it can be done, to allow him to scrutinize those names before they come up. He is then prepared to offer any evidence, or to go before the Comitia Minora if he desires, or to file before the Comitia Minora any information that he may have that may make it questionable whether that man is a fit candidate for membership, or would make a fit member of that organization.

I also noticed this morning that your officers failed to read the names of these applicants, together with those of their vouchers before they were handed over to the Board of Censors. Sometimes it may be impossible to get these in before your programme is issued, and in that case I think they should be brought publicly before the Association, and especially should the vouchers' names be read; for I have had numerous experiences where application blanks similar to these have been filed, and all in the handwriting of the applicant. He has simply misinterpreted the word voucher, and thought it was some one that should recommend him, and he has used whatever name he saw fit. That likewise gives a chance for the voucher, if his name has been used under a mistake, or has been used fraudulently, to intercede before it has gone so far as to elect that man to membership. But, coming back to the original question, it seems to me that you could better solve this question, and likewise make it reach farther—make it reach backward some distance, if you want to—if you find that men have emanated from these schools at such times as they were not doing work that would bear your approval, and upon whom you have placed the seal of your membership, which, of course, carries with it the recognition of that school. I thank you for the privilege of speaking on this point.

*The Chair.*—Are there any further remarks upon the subject of the membership candidates in question?

*Dr. Morris.*—I would beg leave to withdraw my motion, and which has just been discussed, and ask the privilege to bring the matter up later in the form of a resolution, which I would like a little time to prepare. I desire to do this simply to put the matter in a little different form than I have it.

*The Chair.*—As we all know, we have Dr. Hoskins with us. Dr. Hoskins is Secretary of the United States Association, and being a member of that Association myself, I know that he would like to say a few words by way of inducing those of us, who are not members of that Association, to become members thereof ; and I therefore ask that he be given the privilege of the floor a few minutes to speak in behalf of that Association, as our members are all here now. We may not get them again.

*Dr. Hoskins.*—I feel that I have already trespassed too much upon your time. You all know that I am interested in the success of the United States Veterinary Medical Association, but I think it becomes the duty of every member of the profession, who is really interested in the advancement of his calling and in the science in this country, to associate himself with the national organization. It is only by having a strong working body in every centre of the country that we can make our voice heard with any degree of force upon any public question, and surely the questions that are before the veterinary profession to-day are of such grave importance, that our Association should be continuously agitating them and continuously speaking out relative to those important matters. The very question of the veterinary profession of the United States, relative to the past and present actions of the Bureau of Animal Industry of this country, is of sufficient importance for every member in the land to be heard upon, and there is only one way in which the profession may secure any results in that direction, and that is, by unity of action.

Individually, no matter how much we may agitate it in the centre in which we live, and among the people with whom we daily associate, we never have the same force as when it proceeds from a body of men who represent the entire profession of the country. It is then taken notice of, and falls with peculiar force upon those who are responsible for these shortcomings and these faults that are constantly creeping in in connection with the questions that involve the integrity of the profession, its success in the future, and its importance as a factor in the country, in enriching it in so many ways that fall within our line. I therefore appeal to the members of the New York State Association that they should in larger numbers associate themselves with the national organization, and give to it their sympathy and their help in treating and disposing of the grave responsibilities that rest upon the shoulders of that organization. I am willing to concede that the past record of the Association is not one that might wholly commend itself to you ; it is open probably to the gravest criticism, and all the result of carelessness in management and direction, not from any lack of sincerity on the part of the members of the organization, not by design, or anything of that kind, but just from carelessnes in the management of the affairs of the organization. It cannot be said of it to-day, but it can be well said of it to-day that its recognition is sought and desired by every veterinary college of North America. Never was there such agitation and such uneasiness and such anxiety among the various colleges of North America as there was prior to the Boston meeting of last year, when we had up before the profession of the country a proposed alteration of our by-laws that would demand of the future members of that organization graduation from colleges having a curriculum of not less than three years of six months each, legally organized and chartered, and upon whose staff of professors there shall be at least four veterinarians. I was continually importuned for information as to what was likely to be the action of the Association, by those interested in the various veterinary schools of North America, and always replied to them that it was my hope that the amendment would be adopted. It was adopted, as you well know, and in less than two months, in less than one year, one of the leading two-year schools of the country stepped up to the three-year standard. It only shows what an organization may do when it becomes aggressive. It cannot be aggressive unless it has aggressive men in it: and I think there is a large amount of good material—I have seen much to-day—that I hope may be soon associated in active work with the United States Veterinary Medical Association. I hope as many of you as possibly can will be present at our Congress in Chicago commencing next Monday, or, rather, Tuesday, proper. There will be subjects considered and treated there which certainly will interest and instruct you all, give you brighter hopes for the future of the veterinary profession of this country, imbue you with a stronger desire to see that it assumes the level that it should, and that it is worthy of; for there is not a single profession in the entire land upon which rests responsibilities so grave, nor upon which the public are so wholly dependent for health, for happiness and for wealth. And with these few remarks, I hope I may not leave Buffalo at this time without having in my pocket a number of applications. To be sure that there would be no fault on my part I brought a number of blanks with me, and I should be very glad to have a number of your names to carry with me to Chicago. (Applause.)

*The Chair.*—Is there anything to be offered under the head of new business?

*Secretary Hinkley.*—Mr. President, and Gentlemen: Since we last met one of our members has been called away by death, Dr. Kirk. All of you who helped to organize this Society, and all who are charter members will well remember Dr. Kirk as one of our most enterprising and enthusiastic members. Although only a young man, a graduate of the Ontario College, he started in with us like an old member, and there was nothing that would advance the Society, nothing to help us along, that he could do that he would not do. He was only too glad to do anything. He exerted himself, devoting both his time and his money to the interests of the Society. He worked up a good practice at Niagara Falls, but unfortunately was carried away while yet a young man. I would like very much to see an expression of our regret embodied in the form of resolutions, to be presented to his widow and placed on the minutes of the meeting, as we have been in the habit of doing heretofore. I think that it is a tribute that we should all be glad to pay to the memory of poor Dr. Kirk, who was one of our most sincere members.

*Dr. Chase.*—I move you, Mr. President, that the Chair appoint a committee to draft resolutions on the death of Dr. Kirk.

*Dr. Huff.*—I second the motion. Carried.

*The Chair.*—I will appoint as such Committee Drs. Chase, McMullen, and Willyoung.

*The Chair.*—If there is nothing further to offer under the head of new business we will proceed to the consideration of the report of the Committee on Legislation.

Dr. Morris then read his report (see appendix) and added:

I therefore submit the same as Chairman of that committee. It seems as if it was a pretty hard story to tell you who are residents of the Imperial State of the Union, whose Legislature is held in one of the most colossal structures ever dedicated as a hall of legislation, as a place where equity and justice and equal rights are supposed to be dispensed for the benefit of the Commonwealth, and yet, notwithstanding all that, you could not get that bill through that Committee for less than five hundred dollars. Not a cent less—simply because the campaign cost a vast amount of money, and the boys that won in the race had got to settle! I am ashamed as an American citizen, and a citizen of New York State, to have to make such a report, but it is God's living truth, disgraceful as it is.

Another thing in regard to this matter. I was informed by a gentlemen in the eastern counties that a bill was to be presented to amend the act of 1886, which gave authority to all those who had practiced three years to go and sign the register in the County Clerks offices, and upon paying a fee of $2.00 they would become qualified to practice as veterinary surgeons. I had the pledges of three gentlemen, two Democrats and one Republican,—it isn't necessary to name the political complexion of members, so far as that goes,—that that bill should not be allowed to come out of the Committee ; and after it had come out of the Committee and gone through rapidly, I went to the Republican. I said, " Well, you are a fine fellow ; what is the matter with you ? Why didn't you stop that measure ?" " Well, you go to Mr. So-and-so, perhaps he can tell you." " Well," he said, " it was sneaked through and I couldn't stop it, that was all there was of it ; and the men got it that settled ; you should have been here with your money." Now, that is what I was told in plain English ; and there is no bones made about it either. I went away disgusted. The bill was passed and signed by Gov. Flower. Gov. Hill refused to sign any more such bills ; he said so, and had the honor to tell us so, that that bill should not be signed again as an amendment to that original bill. Of course Gov. Flower was under no obligations to Gov. Hill for any promise he had made : so he signed the bill.

*The Chair.*—Gentlemen, you have heard the report of the Committee on Legislation. What is your pleasure ?

*Dr Bell.*—I move that the report be accepted. Seconded ; Carried.

*The Chair.*—The next order of business is the report of the Committee on Constitution and By-Laws.

*Dr. Morris.*—Mr. President, I was nominated as the Chairman of that Committee also, and these by-laws as prepared for your consideration are the result largely of one man's opinion. However, they are open for discussion and amendment ; and the more I read them over the more I see their defects. But, Dr. McLean of Brooklyn, while he was unable to give me all the necessary assistance I desired, nevertheless did very nicely in furnishing me with copies of by-laws from other state organizarions, and Dr. Hoskins has given me very valuable service indeed, and we have endeavored to formulate for your consideration a set of by-laws which would meet the exigencies surrounding our Society, varied as they may be. The Secretary has had these proposed by-laws printed, and before proceeding to act upon them I would move that we take them up clause by clause, discuss them and pass upon them, and in that manner dispose of them as we go along. This idea of reading them all over and then asking you to adopt them is not practical in my judgement. Seconded ; Carried.

The by-laws were then discussed, article by article, and after revision were adopted as a whole (see appendix.)

*Second Day.—Morning Session.*

The meeting was called order to at 9.30 A. M., Vice-President Wende in the chair.

He said : I have here a copy of an Act which is brought by Dr. Hoskins, relating to Veterinary legislaton for the benefit of veterinarians in the U. S. Army, which he desires to have submitted to you for the endorsement of this Society, (see page 364 ) The subject is open for your consideration.

*Dr. Morris.*—I approve entirely of the Act that you have just read ; and I suppose that inasmuch as this Act emanates from the National Society, what the Doctor wants of this Society is simply its endorsement. Is that correct, Dr. Hoskins ? *Dr. Hoskins.*—Yes.

*Dr. Morris.*—Mr. Chairman, I move you that this Society endorse the Act in question I have friends who are Veterinary Surgeons in the Army, and have corresponded with them and visited them, and I know something of the duties and some of the privileges as well as of their status. It certainly has become a passport to society in this country, as it is in Europe, to be an officer in the Army. The veterinary profession in the Army has been to a certain extent degraded. The first application of the term "farrier" in this country, to say nothing of England, was to the blacksmiths of the army, and they looked after the injuries and the diseases of the horses. But in recent years the Government has been employing qualified veterinarians, who it is the object of this Act to raise to the rank of 2nd Lieut. of cavalry. That rank, as I understand it, gives the veterinary surgeon proper allowance—rations, clothing, house rent, light, fuel, and so on. It is certainly a very good rank, and at the same time elevates the profession to a standing to which they are entitled in the Army. I am very much in favor of the proposed measure myself. Motion seconded and carried.

*Dr. Morris.*—I desire at this time to bring before the Society the subject which was laid over from yesterday's discussion, and which was then in the form of a resolution regarding a couple of institutions teaching the art of veterinary medicine. The resolution I will now submit is as follows :

*Whereas*, since the election to membership in this Society of Drs. Pohl and Modinger important information of a questionable nature is at hand in relation to the institutions from which these gentlemen were graduated ;

*Resolved*, that the Board of Censors reconsider the applications, and that Dr. Pohl, who is a resident of this city, be informed, and requested to appear before the Board of Censors, in defense of this action at this meeting.

Now, it seems to me that it is only fair to give this gentleman an opportunity to appear in his own defense. It was very clearly shown yesterday that we were in danger of casting a stigma and disgrace upon Dr. Kelly, innocent of the real facts in his case, and upon the bare fact of his being a graduate of the New York school. It is possible something of the same nature might exist in the case of Dr. Pohl, and it is only right that we should hear before we condemn these gentlemen, or refuse absolutely to admit them to membership in our Society. Dr. Pohl, I believe, is a resident of this city here, practicing here. He was graduated from the Ohio school, which everybody knows, that knows anything about it at all, to be a snide institution, although I believe it has been remodelled. so to speak, in the last few weeks, or two or three months, and the most objectionable features eliminated. However, inasmuch as the school was a most disreputable institution at the time that we understand Dr. Pohl to have received his degree, it would be well enough, in my opinion, to investigate the matter, that this Society may know the character of the concerns from which gentlemen emanate who come here and knock at our doors for admission.

*Dr. Sutterby.*—Has Dr. Pohl been notified of the action taken ?

*The Chair.*—Yes, sir ; he has been notified to present himself here.

*Secretary Hinkley.*—If no one else desires to second that motion I take pleasure in doing so, at the same time explaining my reasons for such a course. Dr. Pohl is a resident of Buffalo, and although a competitor, we nevertheless feel that every care should be taken to see that justice is done him, and, as Dr. Morris says, we came very near yesterday doing a great injustice to one of our members who had been recently elected. Such might be the case with Dr. Pohl. I do not know, but I feel that in justice to him he should be given the privilege of coming here to defend himself.

You can all understand readily why this action is taken. The Society, as you all know, is trying to elevate rather than degrade the profession to which we belong, and if men are graduated from schools with the reputation this one was yesterday shown to have; certainly we do not want them here. Dr. Pohl, however, attended two courses at the Ontario Veterinary School.

*The Chair.*—I think three, Doctor.

*Secretary Hinkley.*—Three, possibly. I do not know what his reputation was there, or his record ; I do not know why he did not graduate. That remains for him to say. But let us do this thing in a spirit of justice to him. If he is not a man properly fitted for membership we do not want to have him ; if he is, we want him. Therefore, I take pleasure in seconding the resolution offered by Dr. Morris. The resolution was then adopted unanimously.

After a recess of fifteen minutes for conference with Dr. Pohl, the Board of Censors reported :

*The Chair.*—The gentlemen will come to order and we will hear the report of the Board of Censors.

*Dr. Morris.*—The Board of censors have investigated as well as they could in the limited time at their disposal the application of Dr. Pohl, and we have deemed it expedient in justice to him and to the Society to lay the matter over until the next meeting, for the purpose of allowing a more thorough investigation. Therefore if our recommendation be adopted Dr. Pohl will not be voted upon until our next meeting. We should like time for further investigation. I would move that the application of Dr. Pohl be laid over until the next meeting. Seconded ; carried.

*Dr. Somerville.*—Is it necessary to take any action on Dr. Kelly's application.

*The Chair.*—He was elected, and Dr. Morris withdrew his name from his motion regarding the non-election of these members.

*Dr. Somerville.*—Then he is elected a member ?

*The Chair* —He is, yes sir, as I understand it.

*Dr. Morris.*—That is correct. I withdrew all my objections in relation to Dr. Kelly.

*Dr. Chase.*—I second the motion. Carried.

*The Chair.*—Is there anything futher to offer under the head of unfinished buisness or new business?

*Dr. Genung.*—I would like to ask if any members in the room can give me any information in regard to a college or institution in Rochester that graduates veterinary students, or gives them certificates in any way purporting to allow them to practice in the State of New York and in the United States. I recently had a young man come into my office and ask in regard to veterinary colleges, and he said that he intended to attend some college or institution, I could not get much out of him, in Rochester. I asked him why he didn't go to some college that was well known. He said this was cheaper and let them out in a much shorter time. I have not heard of it from any other source, and know nothing about it. I would like to know something about it.

*Dr. Drinkwater of Rochester.*—There is nothing there to my knowledge. I think I had the same young man call on me that called on this gentlemen, asking about the same questions, that Dr. Genung says he asked him. I paid no attention to it because there is nothing of the kind there that I know of.

*Dr. Genung.*—A man practicing a short distance from my place, he is not what we term a qualified man, and this young man who intends taking a veterinary course had been talking with him and corresponding some with him. This person who is practicing said he had been to Rochester, and they had taken particular pains to show him all through this institution, and he said they had it working very nicely, and operating room, dissecting room, operating tables, clinics etc. It was a surprise; I had not heard of any such institution and did not know of it. At the time he told me the terms and all about it, but it was in the Spring and it had dropped out of my mind, a good deal of it, until I came here. It would pay us I think to investigate it if possible, to see what there is of it

*The Chair.*—I am afraid you would find it quite a task to investigate if there is nothing to investigate.

*Dr. Sutterby.*—As regards the certificates that some people possess, the country at large is indifferent as to what those papers contain. I know of a party in my district, in practice who has an army discharge, whether or not it is for cowardice in service I do not know; but he has that framed and suspended upon the wall, and points to that as his veterinary diploma. (Laughter.)

On motion of Dr. Morris an adjourment was then taken to 1.30 P.M.

*Second Day—Afternoon Session.*

The Meeting was called to order at 2 P. M. Vice-President Wende in the chair.

*The Chair.*—The next order of business is communications and correspondence. In the absence of Dr. Hinkley, Mr. Drake has that matter in hand. There are quite a number of letters, mostly letters of regret from members and invited guests who were unable to attend this meeting. Among them I recognize the hand writing of Prof. Smith. There is also one from Prof. Law, and others. It remains with the members present whether we shall have them read or not. They are quite lengthy. If there is no objection, then, we will proceed to the next order of business which will be the reading of papers. I will call on Dr. Morris, who has a paper prepared for your consideration, upon Osteo-porosis.*

A vote of thanks was tendered Dr. Morris for his able paper.

*Dr. Robinson* reported a case of Dr. Blank's, in which a horse he had purged passed a worm some 10 feet in length, of a Species unknown to him. The specimen had been sent East to learn its identity. Dr. Somerville exhibited a photograph of the Parasite in question.

*The Chair*—The time and place of the next meeting will now be in order for your consideration.

*Dr. Hinkley.*—That is an important matter that I think had better be decided upon. There was some dissatisfaction about having this meeting in Buffalo, and while you are all here I hope the matter will be decided to the satisfaction of all the members as far as it can be done. After next January the meetings will be annual hereafter, but according to our laws until that time we are obliged to have another meeting for this year, some time in the month of January.

*Dr. Chase.*—I move you that the Secretary be empowered to call the next meeting at such date as he deems best, between the 5th and 15th of January, in accordance with our by-laws.

*Dr. Kelly.*—I second the motion.

*Dr. Morris.*—Before that motion is put it might be well enough to explain the conditions which necessitate another meeting. Our newly adopted by-laws, after this year, require that the meeting shall be held in September; but inasmuch as the current year is controlled by our old by laws, we are obliged under them to have an annual meeting, and that would come in January, to elect officers and so on;

*This paper will appear in December number of JOURNAL, together with remarks and discussion and from that time on the annual meetings will be held in September. The motion was then carried.

*Dr. Morris.*—Before this meeting adjourns I wish to make a motion, that inasmuch as the profession in this city has so handsomely entertained the members of this Society during this two days' session, we express our appreciation of the courtesies that have been extended to us.

*Dr. Sutterby.*—I second the motion Carried, unanimously.

*The Chair.*—Gentlemen, we have credentials from three more applicants which are to be voted upon by the Society. Their names are : Anderson Corforth of Lockport, N. Y. ; vouched for by Drs. Robinson and Hinkley ; J. P. Thompson of Niagara Falls, N. Y., vouched for by Dr. Genung and myself, and Bernard P. Wende, vouched for by Nelson P. Hinkley and myself. The Board of Censors I think have acted upon them ; names are on the applications.

*Dr. Morris.*—I move you that that the gentlemen who appeared before the Board of Censors last and were considered, be now elected, as they have given satisfactory evidence of their right to become members, Drs. Corforth (?), Wende and Thompson.

*Dr Somerville.*—I second the motion. (Carried.)

The following resolution was then adopted :—That the members of this Society pass a vote of thanks to the proprietor and host of the Genesee for their kind and courteous treatment, and to the Press of Buffalo for the attention paid to us and the generous space granted in the daily papers, one and all.

On mo.ion of Dr. Morris the Society then adjourned subject to the call of the Secretary.

[Dr. Morris' paper, revised by-laws, etc. will appear in the December JOURNAL]

